# A case of effective multi-site radiofrequency ablation of premature ventricular complexes in the left ventricular summit

**DOI:** 10.1093/ehjcr/ytaf106

**Published:** 2025-02-26

**Authors:** Emídio Mata, Sílvia Ribeiro, Lucy Calvo, Víctor Sanfins, António Lourenço

**Affiliations:** Cardiology Department, Hospital Senhora da Oliveira Guimarães, R. dos Cutileiros 114, Creixomil, 4835-044 Guimarães, Braga, Portugal; Cardiology Department, Hospital Senhora da Oliveira Guimarães, R. dos Cutileiros 114, Creixomil, 4835-044 Guimarães, Braga, Portugal; Cardiology Department, Hospital Senhora da Oliveira Guimarães, R. dos Cutileiros 114, Creixomil, 4835-044 Guimarães, Braga, Portugal; Cardiology Department, Hospital Senhora da Oliveira Guimarães, R. dos Cutileiros 114, Creixomil, 4835-044 Guimarães, Braga, Portugal; Cardiology Department, Hospital Senhora da Oliveira Guimarães, R. dos Cutileiros 114, Creixomil, 4835-044 Guimarães, Braga, Portugal

A 71-year-old female with dilated cardiomyopathy and an FLNC gene variant, presenting with a 37.6% PVC burden affecting her cardiac resynchronization therapy, underwent successful radiofrequency (RF) ablation of premature ventricular complexes (PVCs) originating in the left ventricular summit (LVS), where PVCs typically show a right bundle branch block (as in this case) or left bundle branch block pattern, low inferior axis, early transition in V2/V3, longer R wave in lead III compared to lead II, pseudo-delta wave, and a negative aVL, aVR, and lead I^1^ (*Figure 1A*).

**Figure 1:**
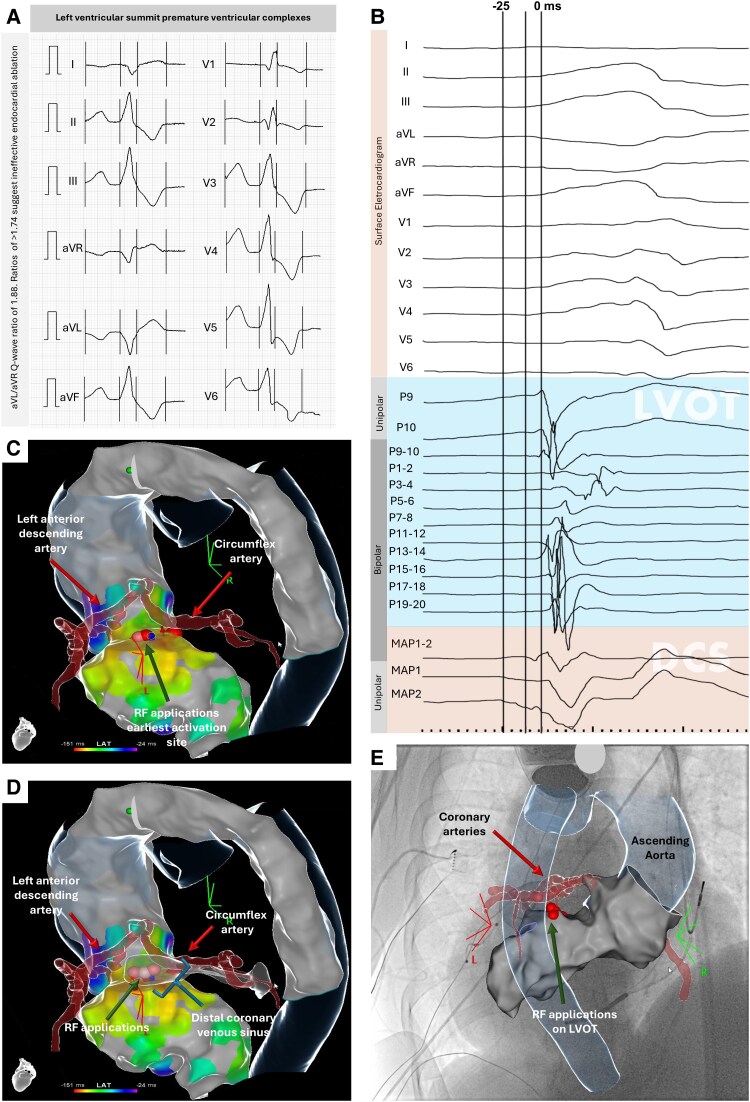
Effective multi-site radiofrequency ablation of premature ventricular complexes in the left ventricular summit. (*A*) Twelve-lead electrocardiogram (ECG) of left ventricular summit (LVS) premature ventricular complexes (PVC). The image shows the electrocardiogram of the left ventricular summit premature ventricular complexes having a right buddle branch block-like pattern, and an inferior axis with aVL/aVR Q-wave ratio of 1.88. (*B*) Surface electrocardiogram with endocardial potentials of PVCs recorded on the left ventricular outflow tract (LVOT) (shaded blue) and on distal coronary venous sinus (DCS) (shaded orange), the latter with the earliest activation recording with a QS unipolar signal pattern (MAP 1, MAP 2). (*C–E*) Radiofrequency (RF) applications (green arrows) were performed on left ventricular outflow tract (*C*) and on distal coronary venous sinus (*D*). These images (*C–E*) demonstrate the proximity of radiofrequency application to the coronary arteries (red arrows).

The complex LVS anatomy, with proximity to coronary arteries and a thick fat pad, complicates ablation.^1^ Preprocedural cardiac CT with electroanatomical mapping Univue software integration was essential for accurate LVS localization (*Figure 1C* and *D*). Without proper preprocedural imaging, the risk of inappropriate lesion placement and coronary artery damage would increase.^2^ Monitoring ST-T changes during RF applications also ensured safety.

Through a retrograde transaortic approach the electroanatomical mapping using the PASO® Module (Carto 3) identified the earliest activation site (*Figure 1B*) under the left coronary cusp, adjacent to the circumflex artery (*Figure 1C*). Radiofrequency ablation was applied with an 8.0F contact force-sensing irrigated catheter (THERMOCOOL SMARTTOUCH® SF) (15 mL/min irrigation; 35–40 W; 15–24 g) with an impedance drop of >10 Ω and an ablation index of 500*f’*, while maintaining a safe margin from the circumflex artery (*Figure 1C*). This resulted in temporary PVC cessation. Due to residual PVCs, three additional applications were made in the distal coronary sinus (DCS) (8 mL/min irrigation; 20–25 W; 27 g), reaching an ablation index of 450*f*. After 30 min, only rare PVCs were recorded (*Figure 1D*). Cardiac resynchronization therapy reassessment 1 month later showed reduction in PVC burden (8.7 PVCs/h) and increased biventricular pacing (98%).

An aVL/aVR Q-wave ratio >1.74 suggest ineffective endocardial ablation.^3^ In this case, with a ratio of 1.88 (*Figure 1A*), multi-site ablation was successful, indicating the epicardial component is possibly more accessible than initially expected.

The management of LVS ventricular arrhythmias has led to the development of various strategies, including epicardial approaches, retrograde coronary venous ethanol infusion, RF via the left atrial appendage, guidewire ablation, bipolar ablation, and surgical ablation.^1^ Recently, focal pulsed field ablation has shown promise. These methods highlight the anatomical and procedural complexities of LVS ablation.

## Data Availability

All relevant data, including the cardiology-related images, are fully displayed and described within the manuscript. No additional datasets were generated or analysed for this study.

## Notes

**Consent:** Written informed consent was obtained from the patient (or their legal representative) for the publication of this case report and any accompanying images.

**Statement of Ethics:** Ethical approval for this study was not required.

**Funding:** None declared.
